# Social marketing and mass media interventions to increase sexually transmissible infections (STIs) testing among young people: social marketing and visual design component analysis

**DOI:** 10.1186/s12889-024-18095-8

**Published:** 2024-02-26

**Authors:** Julie Riddell, Anne Cleary, Judith A. Dean, Paul Flowers, Emma Heard, Zeb Inch, Allyson Mutch, Lisa Fitzgerald, Lisa McDaid

**Affiliations:** 1 MRC/CSO Social and Public Health Sciences Unit, University of Glasgow, Glasgow, UK; 2 Institute for Social Science Research, The University of Queensland, St Lucia, Australia; 3 School of Public Health, Faculty of Medicine, The University of Queensland, St Lucia, Australia; 4 University of Strathclyde, Glasgow, Scotland; 5 Griffith University, Creative Arts Research Institute, Southport, Australia

**Keywords:** Health communication, Health promotion, Social marketing, Adolescents, HIV prevention, HIV testing, STI testing

## Abstract

**Introduction:**

Globally, sexually transmissible infections (STIs) continue to disproportionately affect young people. Regular STI testing is an important public health strategy but remains low among this age group. Raising awareness of testing is an essential step and requires effective interventions designed for young people. To inform the development of effective interventions that promote STI testing among young people, we conducted a systematic literature review to describe the social marketing and visual design components commonly found in STI testing interventions and explore associations of these components with intervention effectiveness.

**Methods:**

We used a systemic review methodology to identify peer-reviewed articles that met pre-defined inclusion criteria. Social marketing and visual component analyses were conducted using structured data extraction tools and coding schemes, based on the eight key social marketing principles and 28 descriptive dimensions for visual analysis.

**Results:**

18 studies focusing on 13 separate interventions met the inclusion criteria. Most interventions used photograph-based images, using conventionally attractive actors, positioned centrally and making direct eye contact to engage the viewer. The majority of interventions featured text sparingly and drew on a range of tones (e.g. serious, humorous, positive, reassuring, empowering and informative) and three interventions used sexualised content. Four articles explicitly stated that the interventions was informed by social marketing principles, with two explicitly referencing all eight principles. Around half of the articles reported using a formal theoretical framework, but most were considered to have theoretical constructs implicit in interventions materials. Four articles provided detailed information regarding developmental consumer research or pre-testing. All articles suggested segmentation and development of materials specifically for young people. Explicit consideration of motivation and competition was lacking across all articles. This study found that there were some design elements common to interventions which were considered more effective. High social marketing complexity (where interventions met at least seven of the 11 criteria for complexity) seemed to be associated with more effective interventions.

**Conclusions:**

Our findings suggest that the incorporation of social marketing principles, could be more important for intervention effectiveness than specific elements of visual design. Effective and systematic use of social marketing principles may help to inform future evidence-informed and theoretically based interventions and should be employed within sexual health improvement efforts.

**Supplementary Information:**

The online version contains supplementary material available at 10.1186/s12889-024-18095-8.

## Introduction

Globally, sexually transmissible infections (STIs) pose a public health challenge particularly among young people [1–3]. Young people experience higher prevalence and morbidity of STIs [3, 4] and barriers to health care [5]. Despite this, research suggests young people may perceive their own risk to be moderately low [4] or may underplay the relevance of sexual health advice to their own circumstances [3, 4, 6–9].

To make informed sexual health choices young people need to comprehend the risks and benefits that may come with adopting behaviours, such as regular testing. Effectively communicating such messages is a central part of health promotion [10]. Health communication is primarily focused on sharing health related knowledge and combatting misinformation, raising awareness, and ultimately facilitating people to make informed choices about their health and behaviours [11–17]. There is a need to identify effective interventions communicating appropriately targeted, evidence informed, health messages to actively engage people [3, 8, 17, 18]. Positive associations between interventions promoting positive health behaviours and adoption of these behaviours have been found [3, 19, 20]. The rise in digital technologies has resulted in increasing access to information, however, being able to design evidence-informed interventions to effectively communicate sexual health messages remains key [13–15]. Adopting a social marketing lens when developing and evaluating health behaviour interventions may be one way to achieve this [20, 21]. Social marketing is an established approach in health communication [22, 23]; however, in a recent study Cook et al. [24] reported inadequate research, including limited analysis of previous interventions, was one of the most frequently listed mistakes associated with ineffective interventions. Despite the key role that social marketing principles and visual design play within interventions aimed at improving health [25–27], little attention has been given to understanding the specific role of social marketing concepts within STI testing interventions aimed at engaging young people [8], particularly the role of visual designs in promoting behaviour change.

To address this gap, we conducted a visual and social marketing component analysis to identify design components commonly found in STI testing interventions aimed at young people.

### Research questions

1. How have visual design and social marketing been employed in STI testing interventions targeted at young people?
2. How do social marketing complexity and visual design elements map against relative intervention effectiveness?
3. What are the key social marketing and visual design components that should be considered in future STI testing intervention design?

## Methods

### The review

We used a systematic approach consistent with best practice guidelines highlighted by Rethlefsen et al. [28]. This review is nestled within a larger systematic review that investigates components related to effectiveness of social marketing interventions to increase HIV and STI awareness and testing in young people. Data extraction for all aspects of the review were completed concurrently. Quality assessments were completed for all studies against standardised tools for qualitative and quantitative studies [29]. As the majority of studies were assessed as having low to fair validity, a meta-analysis was deemed not appropriate. Details of the current review are summarised below:

### Search strategy

We conducted key searches of six interdisciplinary databases CINAHL, Embase, Scopus, PsychInfo, PubMed and Web of Science for studies published between 1st January 2000 and 29th January 2020, using detailed search strategies and standard MESH terms for young people (e.g. adolescen* OR “teen*” OR “young person”….) and STIs (e.g. “HIV” OR “STI” OR “STD”….) and social marketing/ mass media interventions (e.g. “social marketing” OR “health campaign*”….). Search terms listed in Supplementary File 1 were applied to all databases, subject to any database specific requirements. Social marketing terms were searched within title and abstract fields and database searches were supplemented with hand searches of reference lists and key journals. One reviewer (ZI) screened all articles at title and abstract stage, two reviewers screened all articles at the full text stage (ZI, AC), where consensus could not be reached a third reviewer was engaged (LMcD). Searches were updated to include articles published between January 2020 and August 2021.

### Study selection

Studies in which interventions were aimed at young people (aged 10–25) and sought to encourage HIV or STI testing through non-interactive, visual or auditory means were included. The WHO defines young people as 10–24 years [30]; however, we included up to 25 years because a number of articles reported studies using this expanded age group. Studies were not limited to a specific geographic area but were excluded if not available in English. Studies were only included where outcome measures related to HIV or STI testing OR antecedents of testing (such as raising awareness).

Systematic online searches were conducted to locate visual materials (videos, posters etc.) of the interventions described in the included studies. Additional materials were requested from the authors, with a maximum of three requests made. The quantity of materials sourced required a pragmatic approach. For each individual intervention, materials were grouped according to visual similarity (e.g., where the format of the visual remained consistent, such as buttons and stickers with various coloured backgrounds, but a single phrase or logo repeated across all materials). Google translate was used when we were only able to source non-English versions of materials [31], although this is recognised as a limitation.

### Data extraction

Structured data extraction tools were adapted from a previous review completed by the co-authors [21, 32] and are described below (completed tools are included in Supplementary File 2).

#### Visual analysis

The coding scheme, using 28 descriptive dimensions (Table 1) was based on Kress and van Leeuwen’s social semiotic approach [33] and has been previously piloted [21]. The coding scheme provided a framework for visual analysis, which was then adapted to analyse appearance and compositional aspects of the actor(s), setting and props, the social position of the viewer (e.g. close up shots/ direct eye contact), aspects supporting the visual (e.g. use of text or colour), and the social context of the viewer (e.g. where the intervention was viewed) [34, 35]. Detailed descriptions of the content of images were recorded using the framework as a guide. Finally, the combined effect of multimodal materials was considered, including originality of materials, consistency of the combined modes and resulting tone.

**Table 1:** Visual analysis assessment criteria

Visual components		Examples
1) Technical	Type of intervention	poster, leaflet, video
	Medium (could be more than one)	photo, video, diagram, illustration, 3D letter
	Effects	lens flare, fisheye, flash lighting, special photo processing
2) Reading the Visual	Actor’s appearance	Perceived age, perceived gender, clothing, physical features (e.g. hair colour/ skin tone, muscular tone/ body type), other identifying features (e.g. tattoos, glasses)
	Setting/ Environment	e.g. bedroom/ plain white background
	Props/ Objects	e.g. actor holding a ball
	Form of representation	Narrative (action, transactions, mental/ verbal processes)
		Conceptual (classificational, analytical, symbolism)
	Contact	Demand (e.g. direct eye contact, offer of information, services or goods)
	Social Distance	Intimate (close up), Medium (social), Impersonal (distance)
	Point of View	Engagement, Involvement, Detachment
		Viewer power, Equality, Representation power
	Compositional (Salience)	Information value, Framing, Colour, Focus, Texture, Scale
	Modality	High/ Medium/ Low level of truth to image
3) What supports the visual?	Text	Content, Form (e.g., questions, speech, instructions), Font (colour, tone, weight)
	Logos	Relative size, location, type of organisation, recognisable by audience
	Audio	Music, sound effects, speech?
4) Social Context of Viewing	location of materials	Clinic, public billboard, gay scene venue
	references to visual culture	e.g. use of soap opera style dramatization, e.g. telenovela style when targeting Latin American populations
	Societal norms, stereotypes, stigmas, controversies at play	Challenges the stereotype of who contracts STDs/ Challenges silence/ encourages discussion of topic
5) Overall - combination	Intended/ unintended audiences	e.g. if located in gay friendly setting, Gay and bisexual men who have sex with men may be intended or target but others frequenting venue may also view intervention and so would be unintended audiences
	Originality	Unique, surprising
	Provocation	Fear, humour, warmth, irritation, sexual arousal, incongruity, ambiguity
	Consistency of messages	Images and text match in terms of tone e.g. using of non-smiling actor + serious text vs. smiling actor with light-hearted tone
	Tone	e.g. positive, empowering, informative, serious, humorous

Data extracted were analysed to identify design elements evident across interventions and ‘outliers’, i.e., those design elements which were considered significantly different from other design elements identified. When an outlier was identified, the reviewer (JR) checked for consensus with the researcher who conducted the data extraction (ZI). The relative effectiveness of interventions (see Riddell et al. [36]) was then compared to design elements consistently used across interventions and those considered ‘outliers’.

#### Social marketing principles and complexity

We extracted data from intervention descriptions and visual materials on the nature of intervention, mode of delivery, use of imagery, content, and tone of the visual materials, using eight key social marketing principles [37].

1. Behaviour change focus.
2. Theoretical framework employed in intervention design.
3. Insight driven.
4. Customer orientation (e.g. consumer research and pretesting).
5. Segmentation and targeting.
6. Motivational exchange.
7. Competition (i.e. considers appeal of competing behaviours and uses strategies to overcome these.
8. Marketing mix: the four key factors (product, place, promotion, and price) that should be considered when looking to market a service or product (in this case STI/HIV testing).

Standard social marketing definitions of product, place, promotion, and price were employed and determined to contain 11 components, see Table 2 [37]. Characteristics of the social marketing mix of each of the included studies were reviewed and categorised as representing low or high intervention complexity by one author (JR). High complexity was defined as the inclusion of multiple elements (e.g., the use of a single image across an intervention was defined as low complexity and the use of multiple different images was defined as high complexity). Interventions that met at least seven of the 11 components for complexity (Table 2) were defined as having high overall social marketing complexity. This process was adopted from a previous study [21, 32].

**Table 2:** Defining overall complexity of intervention social marketing mix

Social marketing mix		Low complexity	High complexity
Product	Pre-testing	None	User informed
	Provider†	Static	Interactive
	Content	Single	Multiple
	Frequency and duration	One off/short	Long term
	Imagery	Single	Multiple
	Tone	Single	Multiple
Promotion	Segmentation and targeting	All youth	Tailored
	Modes of delivery	Single	Multiple
Place	Settings	Single	Multiple
Price	Motivation	Absent	Present
	Competition	None/single	Multiple

†Provider– static = delivered by two-dimensional media only, i.e., posters/leaflets; interactive = delivered by varied media requiring participants to engage with content, i.e., banner ad linking to online video

Data extraction was completed by one author (ZI), with a 10% sample also coded by another author (JR). Discrepancies identified were resolved through consensus or through discussion with the wider project team.

#### Patterning of effectiveness

Relative effectiveness was used to categorise each study using the following three categories [36]:

1. Intervention had limited or no effect on testing behaviours or antecedents to testing.
2. Intervention had an indirect effect on testing (e.g. increased antecedents to testing such as awareness).
3. Intervention was indicative of clear behaviour change in desired direction (e.g. increase in uptake of STI testing).

Social marketing complexity and visual design elements were mapped to identify the intervention characteristics more commonly associated with intervention effectiveness.

## Results

The final sample included 18 articles focused on 13 separate interventions (Table 3), with six reporting on adaptations of the Get Yourself Tested (GYT) intervention [38–43]. Of the thirteen interventions, eleven were implemented in the United States of America, three in Australia, two in England, one in Denmark and one in Kenya. All 13 interventions were designed specifically for young people; with 8 explicitly for sexually active [38–42, 44–46].

**Table 3:** Overview of intervention components

Intervention Name	Reference	Location of intervention	Intervention description					Overall visual analysis		
			Focus of Intervention and aim	Target Audience	Type of intervention	Social context (Setting of intervention)	Reported target audience involvement in intervention design process	Visual images available for analysis	Use of actors in images	Tone and content
**Check it out Chlamydia hotline**	[46]	**USA**	**Chlamydia**: (a) increase awareness of personal risk for chlamydial infection; (b) facilitate dissemination of chlamydia knowledge by use of a telephone hot line; and (c) promote care-seeking behavior (report for a chlamydia screening program).	15–25-year-old individuals	mail outreach, a television and radio adverts, a prerecorded CheckIt-Out chlamydia hot line, a staffed chlamydia Options information line, and a free confidential urine ligase chain reaction (LCR) test for chlamydia.	wider community- media/ print	implied	No	Unknown	implied informative/ encouraging
**Chlamydia. Worth talking about**	[48]	**UK**	**Chlamydia**: a) increasing chlamydia testing b) normalise conversations about the transmission of chlamydia c) raise awareness of the risk of untreated infection d) explain the process of diagnosis and treatment.	15–24-year-old individuals	national TV, radio, on-line and poster advertising	wider community- online and print/ media	no	yes	no	Positive. Reassuring. Empowering.
**couldihaveit**	[52]	**Australia**	**Chlamydia**: to increase the number of requests for chlamydia testing	sexually active 15–24 year-olds	Radio advertisements, SMSs, poster advertisements, print advertisements, email advertisements sent via radio station electronic mailing lists, interactive website	wider community AND targeted to venues young adults would attend (pubs, universities, clubs etc.)	no	yes	yes-peer representatives	Provocative. Cautionary. Informative, Sexualised elements
**Get Checked Omaha**	[51]	**USA**	**Chlamydia and Gonorrhea**: reduce rates of chlamydia and gonorrhea among young people 15 to 24 years of age. to communicate that no one is immune to STIs and emphasize the importance of STI testing to decrease perceived stigma and increase the demand for testing.	15 to 24 years of age	conventional (billboards, newspaper ads, radio ads) and digital (pre-roll ads, Facebook ads) mediums, a Website, Facebook, Twitter, and YouTube accounts	wider community- digital and print/ media	no	yes	yes-peer representatives	Various: provocative, fear/ disgust, positive/ normalising
**Get Yourself Tested (GYT)**	[38] [39] [41] [42] [43]	**USA**	**Sexually Transmitted Diseases**: increase STD awareness and perceived risk, reduce fear and stigma, and promote open communication with sex partners and health care providers.	under 25 years of age	television, Web, print, short message service (SMS), and on-the-ground efforts to reach youth with information and link them to testing.	wider community, digital and print/ media	yes- formative research	yes	no	empowerment-based approach; positive framing, anti-stigma; normalizing discussions and testing
**Get Yourself Tested (adapted)**	[40]	**USA**	**Sexually Transmitted Diseases**: to promote and routinize STD testing and treatment	sexually active youth, ages 15 to 25 years	postcard and posters, pins and stickers, online and social media outreach, website	wider community- online and print/ media	yes- formative research and materials testing	yes	yes-peer representatives	Empowering. Informative. Non-stigmatising, sexualised elements
**Gimmie 5 min**	[50]	**UK**	**HIV**: challenged the reader to read the accompanying text, which covered the key issues of a pre-test discussion, and then to make a decision about whether they should HIV test.	those under the age of 25 years old and those of Black or South European origin	posters and credit card-sized leaflets	community gay men’s venues	no	yes	yes-peer representatives	Sexualised elements, Informative, Non-judgemental
**HIV. Live with it. Get tested.**	[44]	**USA**	**HIV**: To change youth attitudes about HIV testing and promote more routine testing practices to health providers with the project goal of improving HIV counselling, testing and care among at-risk youth.	sexually active youth of colour in high seroprevalence communities, particularly heterosexual females and homosexual/bisexual males	palm-cards, advertising (print, radio, and video), a youth-friendly magazine or “’zine”, a video news release	wider community and targeted to sites/ media outlets of relevance to youth	yes- formative research	yes	yes-peer representatives	edgy, cool. Instructive.
**Iknowushould2**	[47]	**USA**	**Sexually transmitted infections (STIs)/ HIV**: improve knowledge about and increasing testing for sexually transmitted infections (STIs)/HIV	Targeted youth with a primary focus on youth 13–17 years old in Philadelphia (intervention also included youth 18–24 years old)	traditional media (print advertisements, t-shirts, radio, hotline), new media (website, Facebook, Twitter, Instagram, YouTube), events, and community outreach and partnership	Adverts and events strategically targeted to locations of relevance to youths	yes-coalition provided feedback on branding and content	yes	yes-peer representatives	Peer, positive, private, anti-stigma
**Man Up Monday**	[45]	**USA**	**Sexually transmitted infections, particularly Chlamydia**: to (a) increase awareness of sexual health and chlamydia testing; (b) motivate students, particularly sexually active men who do not pursue regular sexually transmitted infection (STI) testing, to get tested; and (c) improve the capacity of the student health centre to provide free chlamydia testing and treatment for all students	male students at university campus	Mass e-mails, social-media marketing, website, printed materials, posters, campus television and radio interviews, key chains designed to hold condoms (“keypers”), t-shirts, and shorts	college campus, residential halls, online	No	yes	no	reassuring, empowerment, humour
**TESTme screening service**	[54]	**Australia**	**Sexually transmitted infections**: Advertising TESTme screening service	young people (< 25 years) living in rural areas and healthcare providers	websites, a Facebook page, posters, flyers, business cards, wrist bands and professional development sessions for health nurses, advertisements in newspapers, student diaries and short messages to mobile phones	wider community- online and print/ text	No	No- website only	Unknown	Unknown
**VCT**	[31]	**Kenya**	**HIV**: To increase public demand for VCT Phase 1: to build knowledge of and confidence in VCT services, create links between consumers and VCT centers, and launch the VCT logo Phase 2: targeted urban youth (15–24 years) to encourage testing/ know status Phase 3: establishing a norm to know each other’s HIV status during key life events. Phase 4: overtly discussed HIV and AIDS. It targeted male family decision makers and established couples	Phase 1: 15–39-year-olds residing in urban and periurban communities Phase 2: aged 15-24 Phase 3: young couples Phase 4: male family decision makers and established couples	Signboards with the logo at registered VCT sites meeting quality assurance standards, radio, television, posters, flyers, and signage (billboards, street signs).	wider community	no	yes	yes-peer and celebrity	Phase 2 had specific upbeat focus
**you never know who you’ll meet**	[49]	**Australia**	**Sexually transmitted infections, particularly Chlamydia**: To raise awareness of STI, and to promote condom use and STI testing among young people.	heterosexuals aged 18–25 years in Victoria	print and broadcast media, public and internet advertising, and person-to-person methods (Peer-led education)	wider community and youth-targeted venues	implied	yes	yes-peer representatives	Provocative, cautionary, possibly fear based.
**Intervention name not provided**	[57]	**Denmark**	**Chlamydia**: To recruit individuals for C trachomatis testing by use of mailed home obtained samples	individuals aged 21–23 who lived in Aarhus county, Denmark	Online, press (TV/radio/print) releases/ interviews radio interviews, posters and leaflets	Wider community: Online, media (print/TV/radio), venues targeting youth (e.g. halls of residence/ education centres), transportation	no	No	unknown	unknown- paper implies informative

Below we highlight the results of the visual design and social marketing component analyses, and then describe the patterning by reported study effectiveness. Eleven interventions, for which intervention materials could be sourced, were included in the visual analysis and all 13 interventions were included in the social marketing component analysis (Table 3).

### Visual design analysis

The following visual analysis findings relate to the 11 interventions for which 59 individual visual materials were sourced (Table 3). The majority were sourced online and were adaptations of the GYT intervention. Findings relating to social context were extracted from the 13 intervention descriptions. Detailed descriptions of visual and social context of each intervention can be found in Supplementary file 2.

### Appearance and compositional aspects of the actor(s), settings and props

Ten of the 11 interventions [31, 40, 44, 45, 47–52] used photograph-based images and of these seven featured actors (the ‘doer’ of the action [53]) which the researchers of this manuscript considered broadly representative of the target audience (e.g. viewed to be within the target age range) [40, 44, 47, 49–52], with one combining peer and celebrity images [31].

For the majority of images, actors were considered ‘conventionally attractive’ by the researchers of this manuscript (e.g. able bodied, slim, even skin tone), with only one intervention including ‘less polished’ images and depicting actors that a wider proportion of the population may identify with (e.g. uneven skin tone/ braces) [47]. One intervention used multiple actors, one of whom could be considered to have less even skin and visible spots, which may be an effort to make the image more relatable to a wider and younger audience [50]. Three interventions featured images of actors considered explicitly sexual, e.g. of two young people embracing [40, 52] or of semi-nude actors [50]. Regardless of the mode of delivery, materials were generally composed so that actors were positioned centrally, emphasising the actor/s. An exception to this was one where images were shot using amateur style photography and editing techniques to create images that reflect those the target audience may be used to sharing [47].

Interventions where visual materials did not include photographs of people [45, 48]; instead featured photographs of men’s underwear with either a large fishing hook or illustrated flame emerging from the zipper [45] or photographs of scenes (train station/doctors’ office) with speech bubbles reflecting conversations that may happen within the setting [48].

### Social position of the viewer

Meaning can be inferred from the relative position of the viewer and image, contact and point of view. Interventions generally created a relationship between the actor and viewer that was more social or intimate through using ‘medium’ (‘waist’) shots or close ups. Actors within interventions were generally placed at viewers’ eye-level, thus creating an equal power dynamic. Interventions where videos were part of the image suite used a mixture of eye contact, often reflecting interaction between actor and viewer [52], where still images were used they predominantly used direct eye contact, where the actors ‘look’ could be inferred by the viewer as portraying a range of tones from serious [51] to light-hearted and open [31, 47, 49] or flirtatious [47, 50].

### Supporting the visual

Interventions all used text, located on block colours or patterns, which could be considered eye-catching and served to draw the viewers’ attention to the images. The majority featured text sparingly, which often reflected the format of the material (e.g. posters). Those which included brochures or zines naturally included more text [38, 44]. Whilst all of the interventions used both text and imagery, one intervention placed one main headline (the intervention slogan) on a poster in large bold font across the male actor’s bare chest accompanied by three columns of smaller text containing a large amount of detailed information [50].

### Social context of viewing

Two articles reported the use of a single type of setting for the intervention (e.g., only displayed in gay-friendly venues) [43, 50], and all but three reported using online settings [43, 46, 50]. Seventeen reported using a wider community setting (e.g. public transport/ television, bars) [31, 38–42, 44–52, 54] and ten explicitly reported placement in youth focused community locations or events [39–41, 44–47, 49, 51, 54]. Seven reported using education based settings (e.g. colleges) [31, 39, 41, 45, 49, 52] and three used health care settings (e.g. clinics) [43, 45, 46].

### Combined effects of visual design elements

Whilst subjectively assessed, the tone could generally be interpreted as: serious, humorous, positive, reassuring, empowering and informative across the interventions. Tone and content were generally aligned within interventions and across imagery and text (e.g. more serious imagery was accompanied by text that was informative and/or serious in tone). All were targeted at young people, with one explicitly targeting a heterosexual audience [52]. Whilst being sexually active was implied within intervention imagery it was explicitly reported as a criterion of audience segmentation in eight articles [38–42, 44–46] covering four interventions. Garbers et al. explicitly discussed using a mix of sexualised and non-sexualised imagery, based on user feedback regarding the perceived oversexualisation of queer communities [40].

### Social marketing component analysis

All 13 interventions were included in the social marketing component analysis. Intervention descriptions were the key source of data extraction, with the exception of marketing mix, which was also extracted from visual materials (supplementary file 3). Of the 18 included articles, four explicitly stated the intervention was informed by the eight social marketing principles [40, 41, 44, 52] and two explicitly referenced all eight [55, 56]. As suggested by Stead et al., the use of the social marketing label was not consistently applied across interventions descriptions [23]. Of the six articles referencing the same intervention (GYT) [38–43] only one explicitly labelled the intervention as social marketing [40], whilst another alluded to the evidence of the effectiveness of social marketing interventions without explicitly suggesting GYT was a social marketing intervention [38]. The following findings are presented within subsections relating to the eight social marketing principles. Detailed descriptions of social marketing components and mix can be found in supplementary file 3.

### Theory and behavioural goals

Whilst most articles reported the inclusion of some theoretical constructs, this was often implicit in intervention materials, rather than explicitly discussed. Seven articles reported the use of formal theoretical framework [39, 41, 43, 44, 46, 47, 52]. Three of these articles referred to the GYT intervention and thus reported on the same theoretical frameworks (Health Belief Model and Theory of Planned Behaviour) [39, 41, 43]. Another article reported using both the Media Practice Model and Theory of Reasoned Action [46]. Two articles reported the use only of theories specifically related to social marketing principles or theory [44, 52]. The final article referenced the use of the Integrative Model of Behaviour Prediction [47].

When considering behaviour goals identified within interventions, all studies aimed to increase testing (or screening). Three aimed to increase testing and raise awareness or increase knowledge [45, 47, 49]. Four aimed to increase testing, reduce stigma and normalise communication in relation to testing [38, 41, 44, 51]. Seven aimed to increase testing, reduce stigma and normalising communication in relation to testing, and raise awareness or increase knowledge [31, 39, 40, 42, 43, 46, 48].

### Insight and customer orientation

Four articles reported detailed information regarding developmental research with consumers or pre-testing of visual materials [40, 44, 46, 47]. Development research included using focus groups/ interviews with target audience and key stakeholders during the design phase [40, 44, 46] and the use of focus groups to pilot key intervention messages and images with representatives of the target audience [40, 44, 46]. One article explicitly referred to the intervention as being ‘youth-driven’ and developed through partnership with young people and key stakeholders [47]. Dowshen et al. explicitly describe a series of focus groups and sessions where young people contributed to the development of the slogan, content, types of material and design. During these sessions, participants were provided with factual information regarding STI testing processes and Dowshen et al. imply that this new knowledge influenced the development of intervention messages. Three articles reported the use of formative research to guide intervention materials but provided limited details as to what this involved [39, 41, 42, 49]. Another article reported the use of focus groups to pre-test and refine intervention messages, however it was not clear if this also included testing of images or intervention delivery methods [49].

### Segmentation/ targeting

One of the key aspects of social marketing is tailoring to, and acknowledging the wide range of audiences who are, intentionally or not, exposed to the intervention [37]. All articles reported the use of segmentation and development of materials specifically targeting young people and identified specific cities or regions for intervention delivery. Eight articles reported interventions that were explicitly focused on those who were sexually active [38–42, 44–46], although four of these [38, 39, 41, 42] referred to the same intervention branding (GYT).

Four articles explicitly referred to segmentation and development based on sexuality with three focusing on gay and bisexual men [40, 44, 50], one on heterosexual women [44] and one on heterosexual youths [49]. Three articles referred to targeting a specific ethnicity [40, 44, 50], four referred to men [40, 44, 45, 50] one to women [44], and one reported segmentation based on social-economic status [40]. One study tailored to young sexually active college students, suggested the intervention did not focus on a specific group of young people so to promote a more inclusive message [40]. Articles that reported segmentation by ‘at risk’ behaviours often associated risk with being sexually active [40, 44–46]; whilst three articles also alluded to a specific risk of not testing regularly [45] or the specific burden of HIV infections [40, 44]. Motivations for testing (or not) was not reported by any study in relation to the targeting of interventions.

### Motivation and competition

Different issues that may be disincentives (e.g. stigma/ costs) were implied within many of the articles although authors often failed to present an explicit comparison of perceived/ actual costs and perceived/ actual benefits. However, two articles explicitly considered barriers to testing [44, 45] and reported implementing incentives related to testing either through emphasising increased awareness of one’s health [44] or material incentives such as free testing or ‘freebies’ [45]. The final article [40] discussed barriers to testing that were identified via formative research with focus groups and detailed the adaptations made to the intervention to address these [40].

### Marketing mix

The descriptions of marketing mix (***price, product, place***, and ***promotion***) for the included studies are shown in supplementary file 3.

Price refers to the internal and external barriers that compete with the appeal of an intervention, in other words these are the potential costs of a desired behaviour that users must overcome [37]. Whilst several of the included articles reported costs faced by potential users, either explicitly or implicitly alluding to barriers such as stigma or material costs, strategies to overcome competing behaviours and costs were not explicitly discussed.

Product refers to the combination of the use of branding (e.g. logos), the nature and aim of the intervention, frequency/ duration, intensity, and the content, tone and imagery used. The use of an intervention name, brand or logo was explicitly reported within thirteen articles [31, 39–41, 43–45, 47–52]; analysis of visual materials within the remaining five articles suggested they too included an element of branding [42, 45, 46, 54, 57]. Interventions were delivered for a variety of lengths. Some articles reported either the adoption of intervention ‘phases’ during which messages were adapted [39, 44, 51] or images rotated [50]. Few articles reported on intensity (e.g. length of time or number of times potential users might engage with visual materials). A variety of imagery was used, with photographs and video footage most common (see visual design analysis above).

Promotion and place are closely linked and refers to modes of delivery, for example posters, radio, TV, YouTube, social media or other materials, and the wider setting of *where* these are located, for example online or in venues. Our findings suggested multiple modes were generally employed within each intervention. As reported earlier (see Social context of viewing section), online delivery was used by the majority of interventions, followed by youth focused venues, events or wider community settings. Other settings included educational settings (e.g. colleges), or healthcare settings). The use of television, radio, and newspaper/ magazine settings within interventions was also reported within a variety of articles.

### Effectiveness and design elements

For the eleven interventions that had visual material available (Table 4), six interventions suggested evidence of clear behaviour change in the desired direction [31, 44, 47, 50, 52] including adaptations of the GYT intervention [38–41, 43], with three suggesting only limited or no effect on testing or antecedents of testing [48, 49, 51]. The pattern of visual design elements by effectiveness was complex with elements found in interventions indicative of clear behaviour change also present in interventions deemed less effective; however, we observed the following commonalities. Effective interventions generally used a variety of images with actors who were representative of the target audience in at least one of the intervention materials. These interventions all used methods that brought the viewers’ focus to the text or actor through the use of colour and background stylistic choices. All reported using a broader public setting for the intervention or adaptations of the intervention. The three interventions assessed as using more sexual imagery all reported evidence of clear behaviour change [40, 50, 52].

**Table 4:** Social marketing complexity and intervention effectiveness

Intervention Name	Reference	Effectiveness and Intervention complexity		
		Intervention had limited or no effect on testing or antecedents to testing	Intervention had an indirect effect on testing	Intervention was indicative of clear behaviour change in desired direction
**Check it out Chlamydia hotline**	[46]		low overall complexity	
**Chlamydia. Worth talking about**	[48]**†**	low overall complexity		
**couldihaveit**	[52]**†**			low overall complexity
**Get Checked Omaha**	[51]**†**	low overall complexity		
**Get Yourself Tested (GYT)**	[38]**†**			low overall complexity
	[39]**†**			high overall complexity
	[41]**†**			low overall complexity
	[42]**†**			high overall complexity
	[43]**†**			low overall complexity
**Get Yourself Tested (adapted)**	[40]**†**			high overall complexity
**Gimmie 5 min**	[50]**†**			high overall complexity
**HIV. Live with it. Get tested.**	[44]**†**			high overall complexity
**Iknowushould2**	[47]**†**			high overall complexity
**Man Up Monday**	[45]**†**		high overall complexity	
**TESTme screening service**	[54]	low overall complexity		
**VCT**	[31]**†**			low overall complexity
**you never know who you’ll meet**	[49]**†**	high overall complexity		
**Intervention name not provided**	[57]	low overall complexity		

† Included in visual analysis

Intervention descriptions were mainly used to assess complexity in the use of social marketing mix. Where information was missing from intervention descriptions, intervention materials were used to supplement this assessment (e.g. tone of intervention). High complexity in social marketing mix was identified where interventions met at least seven of the 11 criteria with complexity then mapped against effectiveness (Table 4). It should be noted that because the GYT intervention was reported as adaptations of the same intervention we have assessed complexity independently for each variation of the GYT reported in six different articles included, thus one adaptation may be assessed as low complexity while another assessed as high complexity, depending on the information provided within each study.

Of the less effective interventions (rated limited or no effect), a higher proportion rated low on social marketing complexity (75%). The picture was mixed for those interventions rated as high overall complexity. Whilst five of the eleven studies rated as effective suggested low overall complexity of the intervention, it is interesting to note that this includes adaptations of the GYT intervention [38, 41, 43] which was also assessed as high complexity based on details reported within other articles [39, 40, 42]. Most of the highly complex (and effective) interventions involved customer orientation and segmentation. However, this assessment is dependent on information provided within intervention descriptions, which may not detail the full intervention development process and, thus, may explain differences in assessment of the GYT intervention.

## Discussion

Effective health communication to support the uptake of STI testing is essential for improving sexual health among young people. In this review, we have identified common social marketing principles and visual design features within a range of STI testing interventions, and their potential associations with intervention effectiveness. Thirteen of the 18 articles included in our study reported details suggesting evidence of indirect or clear change in behaviour in the desired direction [31, 38–44, 47, 50, 52]. This supports previous research recognising the tendency for unintended or negative consequences to rarely be reported within peer reviewed literature [24], which limits our ability to accurately identify elements linked solely to effectiveness.

We identified key components used across interventions that form the basis of most of the reviewed interventions, including: standard visual content, high social marketing complexity, and key messages relating to reducing stigma and normalising communication about testing, raising awareness and knowledge, and increasing testing. Our analysis found evidence of visual elements that may be associated with effectiveness including the use of actors who were representative of the target audience, presenting interventions within a wider community setting, and the use of background and colour to draw focus to the actor or text. However, these visual elements could also be found in interventions deemed less effective suggesting that there may not be one set visual design brief linked to successful interventions. High social marketing complexity (i.e., where interventions met at least seven of the 11 components) appeared to be associated with effectiveness (i.e. an increase in STI testing or antecedents to testing), suggesting inclusion of social marketing principals in the design phase (for example the use of pre-testing/ insight driven materials), supports effectiveness. Indeed, an understanding of the target audience, their characteristics, and environmental factors is essential to inform culturally safe and responsive interventions [58–60]. These findings support previous research exploring HIV testing interventions targeting gay and bisexual men who have sex with men (GBMSM) [21] and provides further support for the key role social marketing principles could play in guiding intervention development [58–61].

Despite the focus on promoting STI testing and other safer-sex behaviours, most interventions included in this analysis used images or messages that were non-sexualised. This contrasts HIV testing interventions targeting GBMSM, which appear to be highly sexualised [21]. It is possible that this use of non-sexualised images reflects multiple factors including, age of the target population, mainstream location of intervention delivery, potential societal discomfort around discussing sexual health with young people, and (incorrect) assumptions that doing so may encourage sexual activity [62, 63]. This could suggest societal normative beliefs and misconceptions continue to shape how public health presentations are framing messages [64]. Whilst Garbers et al. [40] alluded to the choice of both sexual and non-sexual images being related to feedback regarding oversexualisation of certain communities; due to limited details regarding image selection, we were unable to determine if more sexualised images were discounted during the pre-testing phase of development in other included interventions.

Without detailed knowledge of the process leading to image selection, we cannot be confident that the most effective imagery was selected, and it is entirely possible that the visual designs, regardless of content, included in the interventions did not resonate with the target population (given few had been pre-tested). It is also worth noting that the majority of the included materials featured text sparingly. This could suggest that the purpose or intent of the interventions may be unclear to the target audience and highlights the importance of co-design and consumer engagement in intervention development [65, 66].

High social marketing complexity seemed to be associated with effectiveness, but few articles explicitly referred to all eight social marketing principles. Consistent with our previous work [21] we identified a lack of detailed descriptions of testing and development of intervention materials. However, where it was reported, it was associated with effectiveness, again, highlighting the importance of co-design and consumer engagement [8, 21]. Only one study explicitly reported a co-design process [47], whilst four alluded to the involvement of young people within testing, or piloting materials or messages [40, 44, 46, 49] or that interventions were informed by formative research [39, 41, 42]. However, the absence of process evaluations or intervention manuals within our review limits our understanding of the intervention development process; principles may have been adhered to within the development process but simply not explicitly stated within the literature identified. As noted earlier, analysis of articles reporting adaptions of the GYT intervention resulted in differing assessments of complexity. We suggest that may reflect inconsistencies within intervention descriptions within literature and emphasises the need for caution in interpretation when intervention manuals are not available.

Extensive formative research has been previously associated with a greater likelihood of impact [8, 23]. Intervention descriptions should provide more detailed information relating to both segmentation and formative research, which reflects both the potential positive and harmful effects that materials may have on specific groups of viewers [67]. Formative, or pre-testing sessions, in combination with meaningful evaluations of intervention acceptability and adherence can feed into co-design processes and if facilitated carefully can empower participants to incorporate both lived experience and prior research findings, thereby developing novel or culturally sensitive ideas for delivery and design which may in turn result in more effective interventions [8, 11, 15, 59, 65, 68, 69]. Our findings suggest that social marketing principles are rarely explicitly reported within intervention descriptions, as has been reported elsewhere [23, 70]. This presents a challenge for others to learn from previous interventions.

### Strengths and limitations

This study applied a robust approach drawing from multiple theoretical perspectives to interrogate the visual design and social marketing components of STI testing interventions for young people. Better understanding these components– and their association with effectiveness– is required to inform intervention development [61]. Previous research has suggested that those developing social marketing interventions may not conduct adequate formative research [24]. By providing an understanding of existing interventions, the current study can provide a foundation for further intervention development; however, there are some limitations.

First, the decision to focus on peer published articles means that we were unable to control for publication bias, the tendency for literature to focus on successful interventions rather than publishing studies where interventions were ineffective or produced negative effects. We also acknowledge that the very nature of the intervention development process, where interventions are developed within a specific cultural context, may limit the transferability to other contexts. This emphasises the importance of better understanding the key visual design and social marketing components within these so that these can inform robust intervention development, including co-design and consumer engagement, across different settings.

Second, the data extraction process is necessarily subjective and the reviewer, for both visual analysis (e.g. relative attractiveness of actors) and analysis of tone, play a role in interpretation. The tools adopted sought to minimise subjectivity, including cross-checks between reviewers to ensure consistency, but cannot eliminate it. The volume of intervention materials required these to be analysed in groups, which is common in semiotic studies, [35], but means subtle differences in design elements may have been missed. Similarly, the order in which materials were analysed may have influenced our interpretation of visual elements. Whilst intervention descriptions were limited with regards to materials, they may have contained information such as the target audience which influenced whether visual materials were coded as being more or less representative of the target audience. Visual materials were coded separately from intervention descriptions, with clear guidelines relating to coding visual aspects only if they could be ‘read’ directly from the visual materials. Cross-checking did help to aid transparency although we do acknowledge this as a limitation, not only in coding of the interventions but also in the resulting analysis and interpretation.

Finally, previous studies have reflected on issues regarding the reliance on the term ‘social marketing’ when evaluating interventions [23, 70]. These studies suggest the social marketing label is not applied consistently within literature and therefore may result in some interventions being mislabelled [23]. Instead, several authors suggest using social marketing principles to understand common elements, or ‘active ingredients’ associated with positive outcomes is essential [23, 24, 61]. Thus, the current study focused on the use of systematic tools to gain an understanding of common components linked to effective interventions rather than a social marketing label.

## Conclusions

Young people are disproportionately affected by STIs and increasing testing remains an important. Materials used within interventions aimed at addressing the lack of STI testing among young people must be fit for purpose and relevant to the target audience and drawing on social marketing principles within the design process can support this. Framing health behaviour messages in ways that are congruent with the culture of the target audience, ensures messages are understandable, meaningful, and effective within the target audience [11]. The use of social marketing principles in the development of interventions in this space could support this and here we have demonstrated that interventions which drew on more complex applications of social marketing principles were found to be effective in impacting behaviour change [10, 11, 20, 21]. However, the lack of detailed reporting of intervention development limits our understanding of effectiveness and further investigations are required.

### Electronic supplementary material

Below is the link to the electronic supplementary material.

Supplementary Material 1

Supplementary Material 2

Supplementary Material 3

## Data Availability

The datasets used and/or analysed during the current study are available from the corresponding author on reasonable request. Intervention materials remain the property of original intervention developer(s).
