# Supplementary material for: Social marketing and mass media interventions to increase sexually transmissible infections (STIs) testing among young people: social marketing and visual design component analysis
Source: BMC Public Health. 2024 Feb 26;24:620. doi: 10.1186/s12889-024-18095-8 (PMC10898181; doi:10.1186/s12889-024-18095-8)
Supplement: Supplementary file 2 — Supplementary Material 2 [file 12889_2024_18095_MOESM2_ESM.docx]

**Supplementary File 1: Search terms and Inclusion criteria used in databases (Embase, Web of Science, PubMed, CINAHL, Scopus, PsychINFO)**

| **Age** | **STI** | **Social marketing/mass media** | **Inclusions** |
| --- | --- | --- | --- |
| "adolescen*" OR  "teen*" OR  "youth*" OR  "high school*" OR  "middle school*" OR  "young people" OR  "young person" OR  "young adult" OR  "youth" OR  "boy*" OR  "girl*" OR  "student*" OR  "minor*" OR  "child*" OR  "pre-teen" OR  “emerging adult” OR  “college” OR  “universit*” | "HIV" OR  "HIV1*" OR  "HIV2*" OR  "human immunodeficiency virus" OR  "acquired immunodeficiency syndrome" OR  "genital herpes" OR  "gonorrhea" OR  "gonorrhoea" OR  "chlamydia" OR  "syphilis" OR  "HPV" OR  "human papillomavirus" OR  "STI" OR  "sexually transmitted infection*" OR  "sexually transmitted disease*" OR  “sexual health” OR  “STD*” OR  “AIDS” OR “HIV/AIDS” OR “venereal infection*” OR “venereal disease*” OR  “sex* behavio*” | "social marketing" OR  "mass media" OR  "campaign" OR  "4Ps" OR  "4P" OR  "marketing mix" OR  "audience research" OR  "mass communication" OR  "multimedia" OR  "marketing" OR  "promotional" OR “health campaign*” OR “health communication*” | 2000-2020  English |

**Note: terms as set out in the table were applied to all datasets subject to any database specific requirements.**
